# Calcium Isotopes in Human Urine as a Diagnostic Tool for Bone Loss: Additional Evidence for Time Delays in Bone Response to Experimental Bed Rest

**DOI:** 10.3389/fphys.2019.00012

**Published:** 2019-01-25

**Authors:** Alexander Heuser, Petra Frings-Meuthen, Jörn Rittweger, Stephen J. G. Galer

**Affiliations:** ^1^Steinmann-Institut für Geologie, Mineralogie und Paläontologie, Universität Bonn, Bonn, Germany; ^2^Institute of Aerospace Medicine, German Aerospace Center (DLR), Cologne, Germany; ^3^Department of Pediatrics and Adolescent Medicine, University of Cologne, Cologne, Germany; ^4^Max-Planck-Institut für Chemie, Abteilung Klimageochemie, Mainz, Germany

**Keywords:** calcium isotopes, urine, renal Ca reabsorption, Ca metabolism, bone loss, bed rest

## Abstract

The calcium (Ca) isotopic composition in urine during bed rest has been demonstrated to be systematically light, indicating a negative bone mineral balance (i.e., bone loss). Here we present new Ca isotope data on urine during the “nutritional countermeasures” (NUC) bed rest study. We analyzed the Ca isotopic composition of 24 h pooled urine samples from seven healthy male subjects during baseline data collection (BDC), head-down-tilt bed rest and recovery. Additionally, we analyzed urine from two follow-up examinations after the regeneration phase. We observed a change in Ca isotopic composition during the bed rest phase, indicative of bone loss with a time delay of 10 to 21 days. We also observe that the Ca isotopic composition of urine is strongly dependent on the individual Ca metabolism and varies between subjects. We relate this individuality in Ca metabolism to differences in the amounts of Ca being recycled in the kidneys. Previous studies have shown that the more Ca is reabsorbed in the kidneys the more enriched the urine becomes in heavy isotopes of calcium. The Ca isotopic composition of urine is thus modified by more than one process and cannot be used in a straightforward manner to monitor net bone mineral balance. To overcome this problem, we propose a new baseline approach for using Ca isotopes, which effectively cancels out the effects of individual renal Ca reabsorption. This allows us to detect bone loss in patients without ambiguity by combining measurements of the Ca isotopic composition of urine and daily Ca excretion rate and comparing these to data collected on healthy individuals with a normal steady-state bone balance.

## Introduction

Living under microgravity conditions in space leads to significant changes in the human body and affects human physiology. For example, body fluids are shifted toward the upper part of the body, and mechanical unloading of muscles and bones occurs in the lower extremities. The mechanical unloading of bones leads to bone loss, which is a critical issue for astronauts and especially for those on long-duration space flights. Many of the physiological effects of microgravity-induced changes can be simulated on Earth by a head-down-tilt bed rest study (HDTBR) – namely, a prolonged bed rest at a 6° head-down-tilt position (Rittweger et al., 2006). This is of great interest regarding calcium (Ca) metabolism, since HDTBR drives the net bone balance of the body out of equilibrium.

Calcium plays an important role in many physiological processes such as blood coagulation, synaptic transmission, muscle contraction and cell apoptosis. In order to maintain the functionality of physiological processes involving Ca, the Ca concentration in blood must be held constant within small limits (Ca homeostasis). Three organs play an important role in Ca exchange with the environment: (1) the gastrointestinal tract where Ca is absorbed from the diet, (2) the skeleton which is the main reversible Ca pool in the body, and (3) the kidneys, which regulate the excretion and recycling of calcium from blood. The interaction of these three organs is controlled by hormones (calcitriol, parathormone, and calcitonin) which are secreted in response to the Ca and phosphate concentrations in blood serum (Weaver and Heaney, 2006).

In the past few years, several studies have investigated the use of Ca isotopes as a non-invasive tool for detecting bone loss. During Ca transport in the body, Ca isotopes are fractionated. This was first shown in a pioneering study by Skulan and DePaolo (1999) who examined systematically the Ca isotopic composition of soft and mineralized tissues in vertebrates. One important result they found was that mineralized tissue is enriched in light isotopes of Ca (isotopically light Ca) compared to that of blood. During bone formation Ca in blood becomes depleted in light Ca isotopes while, conversely, isotopically light Ca is released during bone loss to the blood without further fractionation. Gordon et al. (2014) successfully used the Ca isotopic composition of blood serum to predict the active state of multiple myeloma (MM) disease in patients. During the active state of MM, bone is destructed and a lowering of δ^44/42^Ca_blood-serum_ was observed, as predicted. The Ca isotopic composition of blood is also transferred into urine via the kidneys, so changes in δ^44/42^Ca_urine_ can also be used to monitor net bone balance in the body (Skulan et al., 2007; Heuser and Eisenhauer, 2010; Morgan et al., 2012). All three of these studies reported that Ca in urine is strongly enriched in heavy Ca isotopes compared to that of bones and blood. According to Heuser and Eisenhauer (2010) this enrichment occurs during Ca reabsorption from primary urine in the kidney. The light Ca isotopes are preferentially reabsorbed from primary urine and thus lead to a relative enrichment of heavy Ca isotopes in secondary urine. For this reason, the use of δ^44/42^Ca_urine_ as marker for net bone balance is potentially hampered by fractionation of Ca isotopes during urine generation, which is strongly dependent on the fraction of Ca reabsorbed in the kidneys (Heuser and Eisenhauer, 2010; Heuser, 2016).

Here, we investigate the response of the Ca metabolism during simulated microgravity by analyzing the Ca isotopic composition of urine. We hypothesized that during HDTBR, Ca isotopes in urine are sensitive for detecting even small extents of bone loss. Additionally, the samples also allow us to gain further insight into Ca isotope fractionation during Ca transport in the human body under the controlled conditions of a clinical study.

## Materials and Methods

### Study Details and Collection of Biological Samples

Seven healthy, male, nonsmoking, test subjects participated in the “nutritional countermeasures” (NUC) bed rest study. Female subject were excluded due to a higher risk of venous trombosis during immobilization (Bleeker et al., 2004). This study was funded by the German Aerospace Center (Deutsches Zentrum für Luft-und Raumfahrt, DLR) and the European Space Agency. The subjects had mean age: 27.3 ± 3.4 years, mean body mass: 78.3 ± 6.6 kg, mean body mass index (BMI): 24.1 ± 1.9 kg/m^2^, and VO_2_max: 39.5 ± 5.4 ml/kg body mass/min. Approval for the study was obtained from the Ethical Committee of the “Ärztekammer Nordrhein” in Düsseldorf, Germany, and was conducted in accordance with the Declaration of Helsinki. Volunteers gave their written consent after receiving detailed information about the study protocol and the resulting risks. An extensive medical record for each subject was taken, while a routine medical examination and laboratory analyses were done to exclude chronic diseases. None of the subjects received any medication.

Subjects were confined to the bed rest facility of the DLR Institute of Aerospace Medicine, Cologne, Germany for two cross-over designed study campaigns, each consisting of 7 days of pre-bed rest (BDC-7 to BDC-1), 21-days of strict six-degree head down tilt bed rest (HDT1 to HDT21) and 6 days of post bed rest recovery (R + 0 to R + 5; phase R1). Additional follow-up visits took place after 14 (R + 14) and 28 days (R + 28) of bed rest (phase R2). During the 21-day bed rest period, subjects received potassium bicarbonate (3 × 30 mmol day^-1^) as a nutritional supplement aimed at preventing an increased bed rest induced bone resorption as the primary outcome of the study. We focussed upon measuring Ca isotopes in the second part of the overall HDTBR study, which involved four test subjects (B, D, F, and H) with dietary supplementation and three without (A, C, and G) during the bed rest period.

During the HDT period, the subjects stayed in bed 24 h a day and all activities (including showering, eating, and weighing) were performed in head-down-tilted-position, not allowing the subjects to raise their heads above 30° from normal. During the non-bed rest phases (adaptation and recovery), the subjects were allowed to walk around in the ward and to do some exercise tests.

Each volunteer received an individually tailored, weight-maintaining diet. The resting metabolic rate was computed by resting indirect calorimetry on the first day in the ward (Deltatrac II MBH 200 Metabolic Monitor). Energy intake was calculated from the resting metabolic rate and an assumed physical activity level of 1.4 for the non-bed rest phases and 1.1 for the bed rest phases. The total energy intake was augmented by 10% to account for diet-induced thermogenesis. Protein intake was 1.2 g/kg BM/day, 30% of calories were supplied as fat, and the remaining energy calories were from carbohydrates. Daily intakes of calcium (1000–1150 mg/day), fluid (50 ml/kg BM/day), sodium (2.8 mmol/kg BW/day) and potassium (100 mmol/day) were also predefined and strictly controlled during the stationary study period. Minerals and vitamins matched the minimum value for the dietary reference intake (DRI, National Academy of Sciences, Institute of Medicine; Food and Nutrition Board, United States). Because subjects were not exposed to the sun, the dietary intake of vitamin D3 was supplemented (1000 IU/day). No caffeine, or alcohol consumption was allowed. All foods were weighed exactly, and each volunteer was asked to consume the complete meal.

Twenty-four-hour urine collection was done during the whole stationary phase period. Continuous urine was collected daily on a void-by-void basis from 7:00 (±15 min) to 7:00 (±15 min) on the following day. Single voids were stored under darkened and cooled conditions and subsequently pooled to 24 h volumes. Several aliquots of the 24-h samples were stored in the freezer at -20°C for later analysis. Samples on day BDC-6, -2; HDT 2, 10, 21; R + 0, + 2, + 5, + 14, + 28 were processed for later analysis for Ca isotopes. After acidifying the urine sample with 2 mol/l HCl, the samples were aliquoted and frozen at -20°C.

Twenty-four-hour urinary bone resorption marker N-terminal telopeptide (UNTX) was determined with a commercially available assay (UNTX: Osteomark^®^, Wampole Laboratories, United States) in the in-house laboratory of the Institute of Aerospace Medicine (Cologne, Germany). Intra- and interassay-variation were as follows: Intra: NTX 1.3%; Inter: UNTX 4.9%.

### Sample Preparation

Urine containing 25 μg of Ca was pipetted into PFA beakers and 1 ml high-purity concentrated HNO_3_ and 200 μl of H_2_O_2_ (Suprapure^®^, Merck KGaA, Darmstadt) were added. This mixture was placed on a hot plate at 120°C for more than 12 h. After chemical digestion, a small aliquot of the solution containing 2 μg of Ca was taken and an appropriate amount of ^43^Ca-^46^Ca double-spike tracer solution was added (Galer, 2007). Calcium was separated from the rest of the sample using standard cation exchange techniques utilizing quartz glass chromatographic columns containing ∼0.7 mL of Bio-Rad AG 50W-X8 (200–400 mesh) resin and 2.0 M HCl as eluent. The calcium fraction was dried, and a portion of about 1 μg Ca dissolved in 5 wt.% HNO_3_ and loaded onto pre-degassed single or double Re filaments. For single filaments, a Ta_2_O_5_-based activator was loaded as well, as described in Heuser et al. (2002). Loading onto double filaments was different: first, 1 μl of 0.01 M H_3_PO_4_ was placed onto the filament and dried down; then the sample solution was pipetted onto the filament and dried down; finally, the filament was raised to a current of 2.0 A for 30 s.

### Mass Spectrometry and Data Reduction

Calcium isotopic compositions were measured at MPIC by thermal ionization mass spectrometry (TIMS, Thermo Fisher Scientific, Triton) in static multi-collection mode. All raw data were corrected for instrumental bias using the double-spike reduction algorithm assuming the exponential fractionation law (Galer, 2007).

All results are reported using the common δ notation for stable isotope variations (Eisenhauer et al., 2004; Coplen, 2011). δ^44/42^Ca values (in per mil, ‰) were expressed according to:

(1) δ44/42Ca=[(44Ca/42Ca)sample/(44Ca/42Ca)reference)−1]×1000

Here, the reference material used for “zero δ” is NIST SRM-915a, in common with other studies. Samples enriched in light Ca isotopes (isotopically “light”) compared to the reference material have negative δ while isotopically “heavy” samples (heavy isotope enriched) have positive δ-values. Note, that δ^44/42^Ca are a factor of ∼2 smaller than δ^44/40^Ca values, a notation often reported in previous work. δ^44/42^Ca can be converted to δ^44/40^Ca by multiplying by 2.05 (see Heuser et al., 2016).

### Statistics

Statistical analysis and bivariate correlations were performed using the R programming language (The R Foundation for Statistical Computing)^1^. Means and single standard errors (1SE) are given throughout the text. *P*-values for correlations were calculated using a *t*-test approach and are regarded as significant when *P* < 0.05.

The primary aim of the study is to examine the temporal evolution of -6° head-down-tilt bed rest on Ca isotopes in urine. To evaluate trends with time we used a linear mixed effect (LME) statistical model. The statistical tests were performed with time as the fixed effect and the subjects as the random effect. Time was initially coded as an ordering factor, and the two BDC measurements from days BDC-6 and BDC-2 were lumped together into one BDC factor level. Homoscedasticity (homogeneity of variance) and normal distribution of residuals were evaluated using residual plots and quantile-quantile plots. Where significant effects of time were revealed by ANOVA, they were further studied by *a-priori* contrasts of the treatment type. Data are reported as best linear unbiased predictors (BLUP) and standard error (SE). The level of statistical significance was set to 0.05.

## Results

Anthropometric data for the seven test subjects are listed in Table 1. Ca isotopic compositions and Ca concentrations of urine as well as the total Ca excreted per 24 h can be found in Table 2.

**Table 1:** Anthropometric data, δ^44/42^Ca (‰), errors (1SE), and Ca flux (mmol/d, F_Ca_) of the subjects from the head-down-tilt bed rest study.

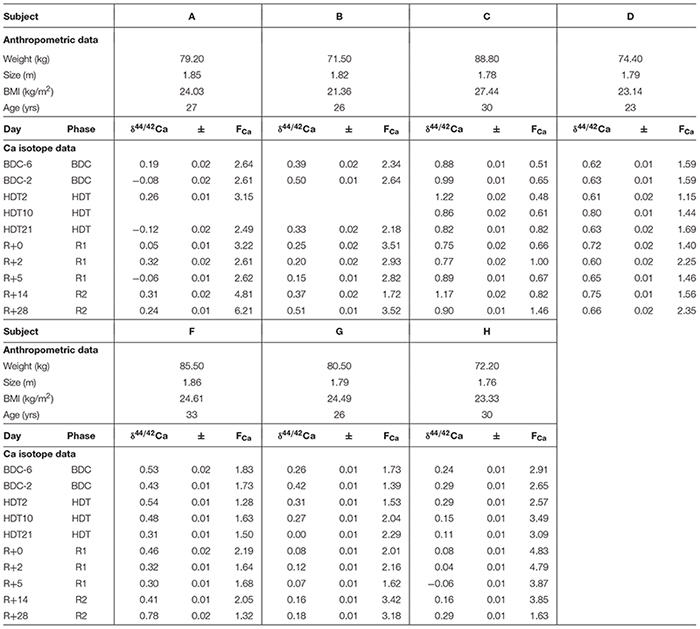

**Table 2:** Best linear unbiased predictors (and their standard errors) as obtained from the linear mixed effect model, and the *P*-levels for the *a priori* contrasts.

Day	δ^44/42^Ca	1SE	*P*
BDC	0.45	0.12	<0.001
HDT02	0.08	0.05	0.1407
HDT10	-0.01	0.06	0.9034
HDT21	-0.15	0.05	**0.0037**
R+0	-0.11	0.05	**0.0344**
R+02	-0.11	0.05	**0.0307**
R+05	-0.17	0.05	**0.0011**
R+14	0.03	0.05	0.5769
R+28	0.06	0.05	0.2551

*Significant time effects have been set in bold face*.

**Figure 1:**
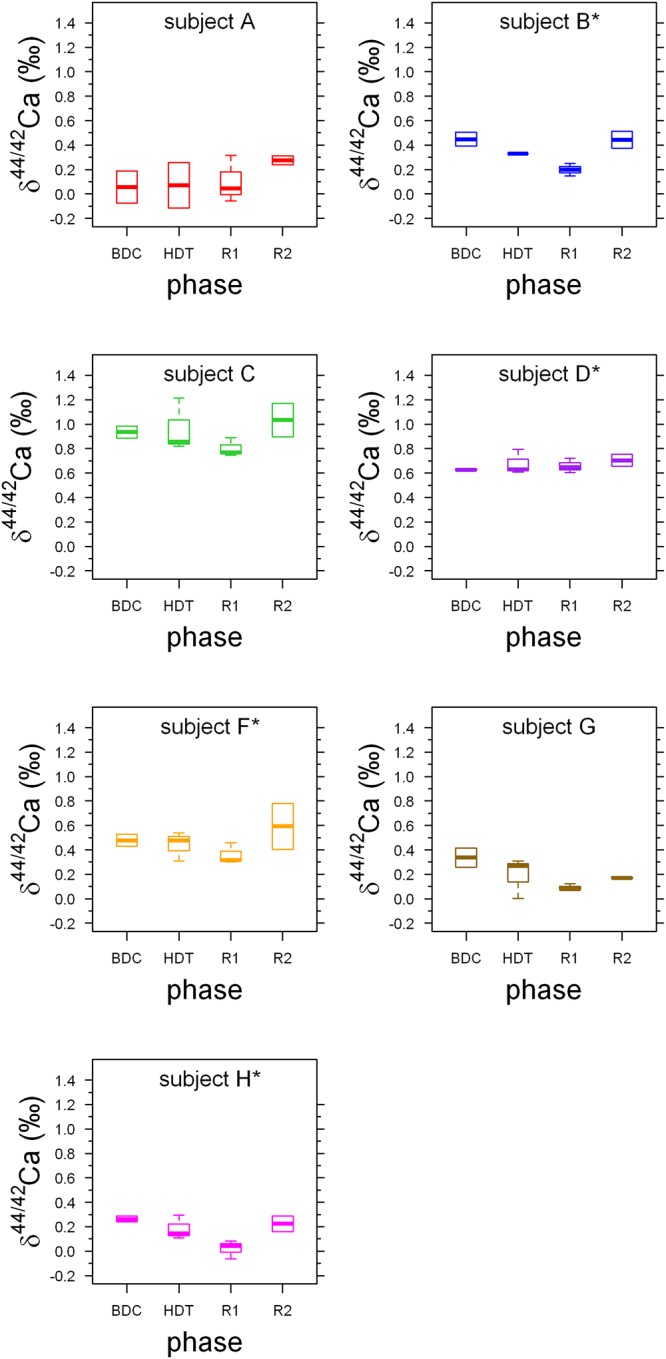
Boxplots of δ^44/42^Ca_urine_ values for each phase for the seven subjects A to H. Stars indicate subjects with KHCO_3_ supplementation. Phases are: BDC: pre-bed rest baseline data collection (BDC-6 and BDC-2), HDT: head down tilt bed rest (HDT2, HDT10, and HDT21), R1: post bed rest recovery (R + 0, R + 2, and R + 5) and R2: outpatient regeneration (follow-up examination; R + 14 and R + 28).

**Figure 2:**
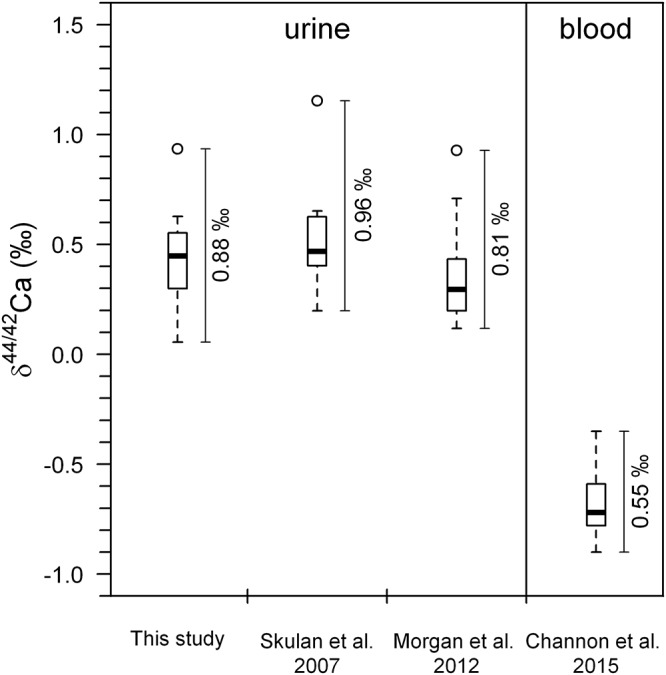
Boxplots of Ca isotopes showing pre-bed rest data (BDC) for all subjects combined from this study (*n* = 7), along with the studies of Skulan et al. (2007) (*n* = 9), Morgan et al. (2012) (*n* = 12), and Channon et al. (2015) (*n* = 12). Data from Channon et al. (2015) and Morgan et al. (2012) were obtained from identical subjects in the same bed rest study. Open circles represent statistical outliers. The span (max–min) in the δ^44/42^Ca_urine_ values is higher than the observed span in δ^44/42^Ca_blood_ values. The data from Skulan et al. (2007) have been recalculated from the original δ^44/40^Ca values. All literature data have been converted to conform to NIST SRM-915a reference material (see Section 3.2).

δ^44/42^Ca_urine_ values cover a wide range from +1.2‰ (subject C, HDT2) to -0.1‰ (subject A, HDT21) (cf. Figure 1). The dataset shows two remarkable features: (1) huge inter-individual differences of up to 0.8‰ exist in δ^44/42^Ca_urine_ (Figure 2) and these differences stay nearly constant throughout the study; (2) statistical testing (Table 2) shows that the secular variation in δ^44/42^Ca is statistically significant (*P* < 0.001). The initial δ^44/42^Ca at the start of the treatment period was 0.45 ± 0.12 (1SE) at BDC; this decreased systematically by 0.15 ± 0.05 (1SE) on HDT21 (*P* = 0.004), by 0.11 ± 0.05 (1SE) on R + 0 (*P* = 0.034), by 0.1711 ± 0.05 (1SE) on R + 2 (*P* = 0.031), and by 0.17 ± 0.05 on day R + 5 (*P* = 0.0011). Notably, no statistically significant change was observed on day HDT2 (*P* = 0.14) or on day HDT10 (*P* = 0.90).

## Discussion

### Individuality of Ca Metabolism

Boxplots of the δ^44/42^Ca_urine_ for each of the subjects A to H (Figure 1) show that the urinary Ca isotopic compositions during pre-bed rest BDC span a wide range and are different for most of the subjects. The maximum difference of about 0.8‰ is observed between subjects A and C. Such huge inter-individual Ca isotope differences have been observed previously for urine and blood samples from other bed rest studies (Skulan et al., 2007; Morgan et al., 2012; Channon et al., 2015) as well as in archeological bones (Reynard et al., 2010). Figure 2 shows a comparison of the observed inter-individual ranges in δ^44/42^Ca_urine_ and δ^44/42^Ca_blood_. The span of δ^44/42^Ca_urine_ in our dataset is the same order of magnitude as that already reported in the literature (Skulan et al., 2007; Morgan et al., 2012).

In their pilot study, Heuser and Eisenhauer (2010) analyzed δ^44/40^Ca_urine_ of a 63-year-old woman suffering from osteoporosis and a 4-year-old healthy boy. A difference of 1.1‰ in δ^44/40^Ca (0.55‰ in δ^44/42^Ca) was observed between their average urinary Ca isotopic compositions. This offset was attributed to differences in the net bone balance of the two subjects. While the woman had a negative bone balance (loss > gain) the opposite was true for the boy. It can be assumed that the subjects A to H of this bed rest study are not very different in their pre-bed rest net bone balance. Thus, it is unlikely that net bone balance could be the main cause of the inter-individual differences in δ^44/42^Ca observed in Figure 1.

The principal finding of this study is that δ^44/42^Ca is systematically lowered during bed rest. However, this lowering is occurring with a time delay of 10 to 21 days. After re-ambulation, δ^44/42^Ca returns back to baseline values again, after a time delay of 6 to 14 days. Similar time delays have been observed for biochemical markers of bone resorption at the onset and termination of bed rest (Armbrecht et al., 2010) Furthermore, bone mineral content of the tibia decreases for another 10 to 20 days after re-ambulation following bed rest (Rittweger et al., 2010) and after unilateral limb suspension (Rittweger et al., 2006).

Based upon the Ca isotope dataset of our study, we can establish that time delays in the bone’s response to disuse and re-ambulation are present on the levels of: (1) the organic matrix turn-over, (2) calcium trafficking between body fluids and bone, and (3) the anatomical bone structure. As hypothesized by Rittweger et al. (2016), we propose that differentiation and migration of osteoclast precursors and of osteoblasts provide the most likely explanation for the time delays observed. If so, this would imply that determination of bone precursor cells may be a more potent driver of bone’s mechanical adaptation than modulation of existing bone cells.

One also needs to consider that differences in dietary Ca isotopic composition (δ^44/42^Ca_diet_) could be the origin of the observed differences in the δ^44/42^Ca_urine_. But since all test persons received a similar diet, inter-subject differences caused by δ^44/42^Ca_diet_ should decrease during the course of our study. However, this is not observed (cf. Table 1); rather, the inter-individual differences stay nearly constant during the whole study period. In addition, it has been shown that the Ca isotopic composition of a diet that is dominated by dairy products (Chu et al., 2006; Heuser and Eisenhauer, 2010; Heuser, 2016) does not vary as much as the 0.8‰ range observed in our study. In summary, δ^44/42^Ca_diet_ probably accounts for only a minor portion of the observed inter-individual differences of δ^44/42^Ca_urine_.

**Figure 3:**
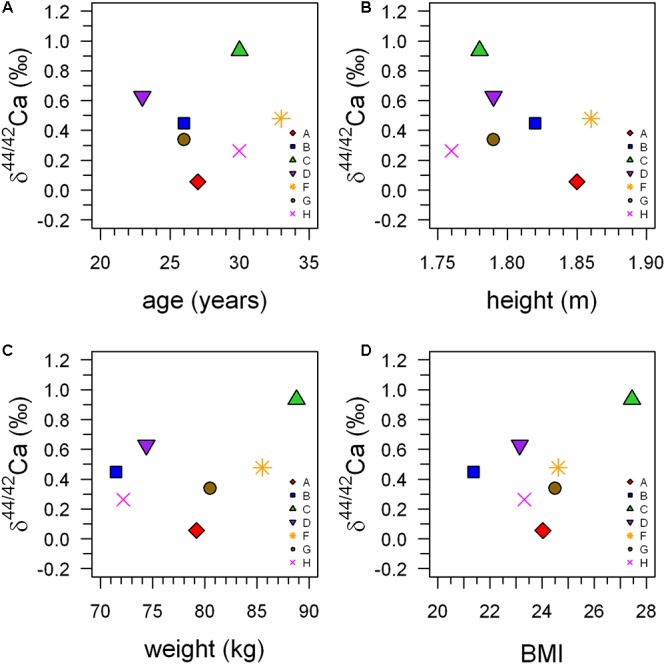
Average δ^44/42^Ca_urine_ during the pre-bed rest phase (BDC-6 and BDC-2) plotted against age **(A)**, size **(B)**, weight **(C)**, and body mass index (BMI) **(D)** of the subjects. This illustrates that there are no statistically significant correlations between δ^44/42^Ca_urine_ and any of the anthropometric parameters.

The observed differences in average pre-bed rest δ^44/42^Ca_urine_ are also not correlated to age (*r*^2^ = 0.11, *P* = 0.858), weight (*r*^2^ = 0.21, *P* = 0.295), body height (*r*^2^ = 0.11, *P* = 0.467) or the BMI (*r*^2^ = 0.26, *P* = 0.239) of the subjects (Figure 3).

It is possible to calculate δ^44/42^Ca_urine_ using a Rayleigh-type fractionation model (Heuser and Eisenhauer, 2010):

$$\begin{array}{l} \delta^{\mathrm{44}/\mathrm{42}}{\mathrm{Ca}}_{\mathrm{urine}}\;=\;(\delta^{\mathrm{44}/\mathrm{42}}{\mathrm{Ca}}_{\mathrm{blood}}\;+\;\mathrm{1000)}\;\cdot \;f_{\mathrm{excreted}}{}^{\;(\alpha -1)}\;-\;1000\;\;\;\;\;\;\;\;\;\\ \;\;\;\;\;\;\;\;\;\;\;\;\;\;\;\;\;\;\;\;\;\;\;\;\;\;\;\;\;\;\;\;\;\;\;\;\;\;\;\;\;\;\;\;\;\;\;\;\;\;\;\;\;\;\;\;\;\;\;\;\;\;\;\;\;\;\;\;\;\;\;\;\;\;\;\;\;\;\;\;\;\;\;\;\;\;\;\;\;\;\;\;\;\;\;\;\;\;\;\;\;\;\;\;\;\;\;\;\;\;\;\;\;\;\;\;\;\;\;\;\;\;\;\;\;\;\;\;\;\;\;\;\;\;1000\;\;\;\;\;\;\;\;\;\;\;\;\;\;\;\;\;\;(2) \end{array}$$

where α is the fractionation factor between primary and secondary urine, δ^44/42^Ca_blood_ is the input Ca isotopic composition of the blood and *f*_excreted_ is the percentage of Ca not reabsorbed in the kidneys, that is collected in the bladder and then excreted. The fractionation factor α is a measure for the amount of isotopic fractionation in an exchange reaction. Assuming the same fractionation factor α between subjects it can be seen from Equation (2) that differences in *f*_excreted_ between subjects have a major influence on δ^44/42^Ca_urine_ and should also be reflected in the amount of Ca excreted via urine. In particular, the more Ca that is reabsorbed from primary urine, the less total Ca that will be excreted, and the higher the δ^44/42^Ca_urine_ becomes. This can be tested since differences in the fraction of Ca being reabsorbed in the kidneys between subjects should be reflected in the amount of Ca excreted via the urine.

**Figure 4:**
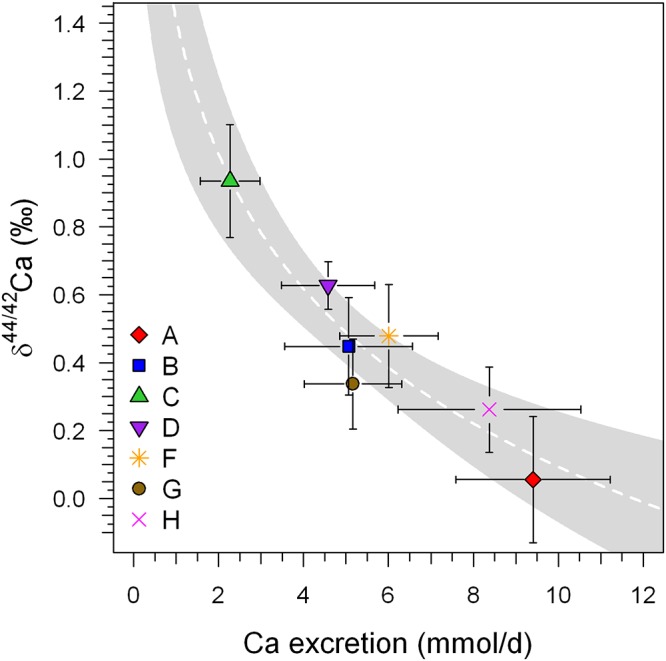
Mean δ^44/42^Ca_urine_ (±1SD) of the BDC (BDC-6 and BDC-2) phase of each subject plotted against the average excreted Ca per 24 h (±1SD). A significant logarithmic correlation exists that is statistically significant (*r*^2^ = 0.884, *P* < 0.001; white dashed line). Such a correlation is typical for Rayleigh-type fractionation such as might be expected during generation of secondary urine in the kidneys. These data were collected before and after the head-down-tilt bed rest period. The gray shaded area represents the 95% confidence band of the logarithmic regression trend.

Indeed, a significant correlation (*r*^2^ = 0.93, *P* < 0.001) does exist between δ^44/42^Ca_urine_ and total excreted Ca (mmol/24 h), as can be seen in Figure 4. This correlation suggests that variable individual renal Ca reabsorption is the main cause of the observed inter-individual differences in δ^44/42^Ca_urine_.

Furthermore, the amount of renal Ca reabsorption is not directly linked to dietary Ca input. The dietary Ca intake between all test persons varied by a factor of 1.2, being only slightly lower than the amounts of individual excreted Ca (factors of 1.3 to 2.3). The differences in the excreted urinary Ca between the subjects varied by, on average, a factor of 4.3 ± 1.3 (1SD). This pattern of higher differences between subjects compared to differences over the course of study for an individual can also be observed in the δ^44/42^Ca_urine_ dataset, which shows smaller intra-individual variation than inter-individual differences.

A change in the fractionation factor during renal Ca reabsorption cannot be excluded. Such a fractionation factor change might plausibly be linked to the amount of cells being involved during transcellular and paracellular transport of Ca in the kidneys, which is ultimately controlled by different hormones (e.g., Mundy and Guise, 1999; Hoenderop et al., 2005; Peacock, 2010). Such a scenario might also change the fraction of Ca reabsorbed, with both effects combining and amplifying the net extent of Ca isotope fractionation in the urine.

### Response of δ^44/42^Ca_urine_ to Bed Rest

**Figure 5:**
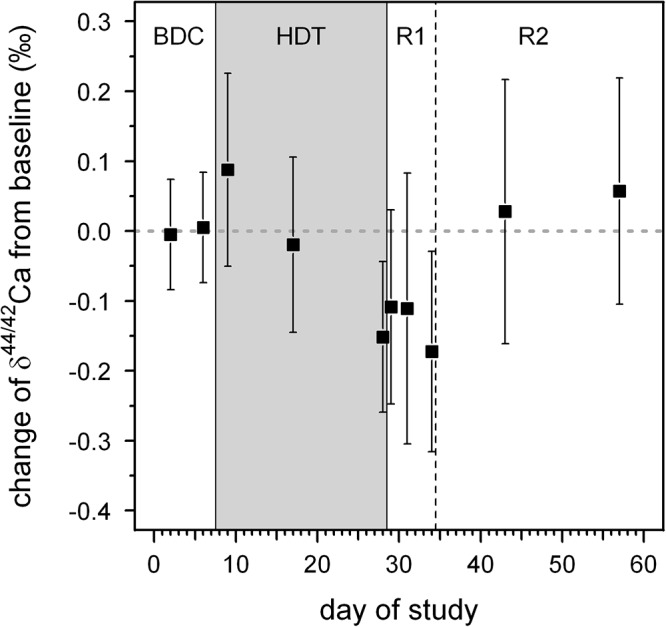
Average changes in δ^44/42^Ca from baseline values for all subjects as a function of day during our bed rest study. The baseline value is represented by the average δ^44/42^Ca of all subjects during the BDC period (BDC-6 and BDC-2). The phases of the study are: BDC: pre-bed rest baseline data collection, HDT: head-down-tilt bed rest, R1: inpatient regeneration and R2: outpatient regeneration (follow-up examinations).

**Figure 6:**
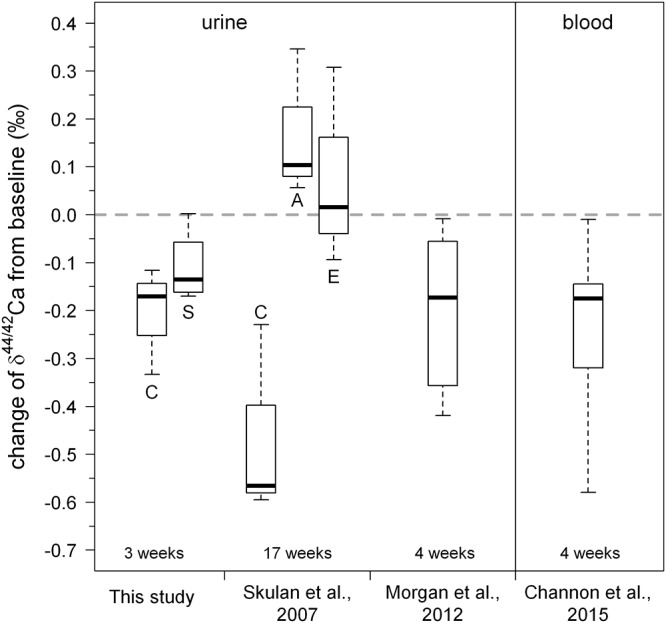
Boxplot of changes in urinary Ca isotopic composition obtained during our study. For comparison, the data of Skulan et al. (2007); Morgan et al. (2012), and Channon et al. (2015) are also shown. Overall, the changes observed in the study are in good agreement with those found in previous studies. A small but statistically insignificant difference between the control group (C) and the KHCO_3_ supplemented group (S) can be seen. Letters for the Skulan et al. dataset correspond to: (C) control group, (E) exercise group, and (A) alendronate supplemented group.

In general, δ^44/42^Ca_urine_ decreases during the bed rest phase (Figure 5). This change in δ^44/42^Ca_urine_ is fully in accord with isotopically “light” Ca being released from bones during bone resorption and, indeed, the changes during bed rest in our study match those reported in the literature (Skulan et al., 2007; Morgan et al., 2012; Channon et al., 2015; see Figure 6). We found a change, on average, of about -0.15‰ over the time of bed rest, while Skulan et al. (2007) reported a change of -0.48‰ (control group, 17 weeks of bed rest) and, similarly, Morgan et al. (2012) found -0.2‰ (4 weeks of bed rest). The differences in δ^44/42^Ca_urine_ shifts during bed rest periods observed between studies can most likely be attributed to the different length of the bed rest phase in each case.

Our Ca isotope dataset shows a small difference between the group of subjects with and without supplement of potassium bicarbonate (KHCO_3_), although this is statistically not significant (Figure 6). Specifically, the mean change of δ^44/42^Ca_urine_ from baseline is -0.21‰ for subjects without KHCO_3_ supplement, while it is -0.11‰ for those who took the supplement.

Up to now, changes in δ^44/42^Ca_urine_ have been interpreted to be the consequence of bone resorption only. Support for this explanation comes from (1) the data of Channon et al. (2015) who, like in the present study, found a decrease in δ^44/42^Ca_blood_ during bed rest, and (2) from Skulan et al. (2007) who reported a correlation between δ^44/42^Ca_urine_ and changes in conventional serum biomarkers for bone resorption. Other physiological effects during bed rest that might lead to changes in δ^44/42^Ca_urine_ have not yet been fully investigated, however.

For example, assuming a constant amount of Ca transported from the intestine into blood along with prolonged bone resorption during bed rest, the Ca concentration in blood must increase and will be regulated by an increase in overall Ca excretion. But, in order to increase Ca excretion, the fraction of Ca that is reabsorbed in the kidneys (*f*) must decrease. In terms of Ca fractionation this will result in lower δ^44/42^Ca_urine_ as increased amounts of Ca are being excreted (cf. Figure 4). A decrease in the fraction of Ca being reabsorbed from 98 to 97% would cause a lowering of δ^44/42^Ca_urine_ by about -0.1‰. Thus, the shift in δ^44/42^Ca_urine_ during the bed rest phase can be explained by either the addition of isotopically “light” Ca from bones to the blood or, alternatively, by a change in the renal Ca excretion factor (f) or, more probably, the combined effect of both.

The observed inter-individual differences in δ^44/42^Ca_urine_ and corresponding δ^44/42^Ca_blood_ from the studies of Morgan et al. (2012) and Channon et al. (2015), respectively, support a changing excretion factor (*f*) during bed rest. While the inter-individual differences (max δ^44/42^Ca–min δ^44/42^Ca) are about 0.85‰ for δ^44/42^Ca_urine_ at the end of the bed rest phase, the reported shift in δ^44/42^Ca_blood_ is smaller (0.46‰).

On the other hand, an increase in the concentration of free Ca in the blood triggers a serum parathyroid hormone (PTH) concentration decrease (Mundy and Guise, 1999). Consequently, the decrease of PTH concentration then results in a decrease in intestinal Ca absorption and a lowered renal Ca reabsorption (Mundy and Guise, 1999).

### Comparison of δ^44/42^Ca_urine_ With NTX

**Figure 7:**
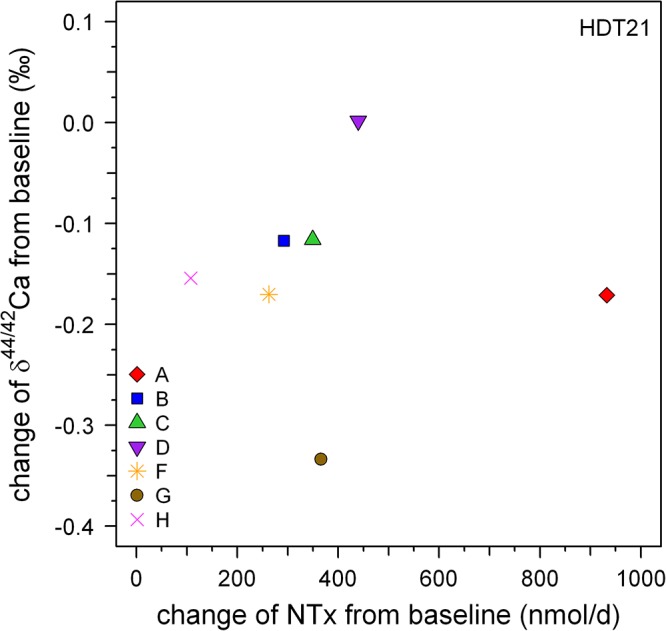
Plot of changes in Ca isotopic composition versus those in NTX excretion, both from relative to baseline at the end of the bed rest phase (HDT21). Qualitatively, both biomarkers show bone loss but there is no strong correlation evident between them.

Serum NTX (n-telopeptide cross-links) is a frequently used biochemical marker for bone resorption (e.g., Iba et al., 2008). Skulan et al. (2007) reported changes in NTX alongside δ^44/42^Ca_urine_ in their samples. At first glance their data suggests a correlation between NTX and δ^44/42^Ca_urine_ since mean δ^44/42^Ca_urine_ decreases during bed rest as mean NTX increases. This qualitative correlation suggests that changes in δ^44/42^Ca_urine_ during bed rest are linked to increased bone resorption. Such a qualitative correlation between δ^44/42^Ca_urine_ and changes in NTX is also seen in our dataset. Relative to pre-bed rest values, the mean change in δ^44/42^Ca_urine_ post-bed rest is -0.10‰ while the corresponding change in NTX is 393 (nmol/d). But a closer look at the pooled data shows that there is in fact no statistically significant correlation between δ^44/42^Ca_urine_ and NTX (Figure 7; *P* > 0.99), nor for any of the subjects individually (cf. Table 2).

### A Promising New Baseline Approach

Based on our data and those from previous studies, bone loss cannot be detected unambiguously using δ^44/42^Ca_urine_ if the (steady state) pre-bone-loss baseline δ^44/42^Ca_urine_ of the patient is unknown. This hampers the easy application of δ^44/42^Ca_urine_ as a diagnostic tool for detecting disease related to bone loss, such as osteoporosis. Here, the main problem seem to be in the unknown amount of Ca being reabsorbed in the kidneys for each patient. But on the other hand, using δ^44/42^Ca_urine_ it should still be possible to monitor the success of an on-going osteoporosis therapy aimed at reducing bone resorption.

**Figure 8:**
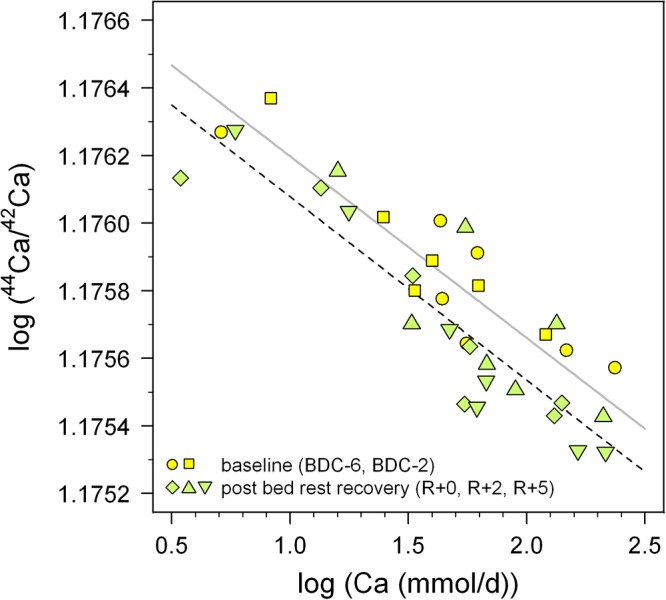
Log-log plot of ^44^Ca/^42^Ca in urine versus daily-excreted Ca (mmol/d) for the pre-bed rest BDC data (BDC-6 and BDC-2, yellow squares) and directly after the bed rest phase (R + 0, R + 2, and R + 5, green diamonds). Both sets of data show negative correlations with trend lines indicated (BDC, gray; R + 0, black dashed), with the after bed rest data shifted systematically toward lower ^44^Ca/^42^Ca, i.e., more negative δ^44/42^Ca. This vertical offset likely reflects bone loss during bed rest independent of changes arising from variable renal Ca reabsorption in the kidneys.

In order to overcome this problem we propose a new baseline approach intended to reduce the unknown renal Ca absorption effect on δ^44/42^Ca_urine_. In a log-log plot of ^44^Ca/^42^Ca_urine_ versus the amount of excreted Ca per day (Figure 8) two different negatively sloped trends can be seen, for the adaption (BDC) and bed rest (R + 0, R + 2, R + 5) phase. The BDC samples follow a linear trend (*r*^2^ = 0.780, *P* < 0.001) with a slope of -5.38 × 10^-4^ and a *y*-axis intercept of 1.177, while the bed rest samples linear trend (*r*^2^ = 0.790, *P* < 0.001) has a slope of -5.43 × 10^-4^ and *y*-axis intercept of 1.176. Although both trends are not strictly parallel to one another, they remain basically parallel within the range of excreted Ca measured. The offset between the two trends seen in Figure 8 indicates that the Ca isotopic composition for any given Ca excretion rate is always isotopically “lighter” at the end of the bed rest relative to the BDC baseline.

Thus, we suggest that a combined measurement of daily Ca excretion rate as well as δ^44/42^Ca_urine_ may be sufficient to implicate patient bone resorption, if the data lie below the BDC trend when plotted in Figure 8. For the time being, this inference must be considered tentative, pending better definition of the BDC line in Figure 8 from future studies; this can be done relatively easily, however, since it just involves measurement of these two parameters in healthy individuals whose Ca balance is at steady state.

We used the R1 data here, as they should be the most affected by the bed rest phase. Based on Figure 6 it seems justified to assume that the shift from the baseline trend would be even more pronounced during longer periods of bed rest. Figure 6 also shows a small but not significant difference between the control group (C) and the supplemented group (S). While the control group defines a linear trend shifted parallel toward lower δ^44/42^Ca, the supplemented group defines a trend with a lower slope and higher y-axis intercept. Currently, we do not have a good explanation for this – it might be related to the supplement of KHCO_3_ but may also be simply a statistical artifact due to the limited number of subjects in each group (n_C_ = 3, n_S_ = 4).

One should keep in mind that the data currently available, here and elsewhere, are far too few in number to define a statistically meaningful BDC δ^44/42^Ca_urine_ versus excreted Ca rate baseline. Nevertheless, it does reveal a promising way for canceling out the patient-specific effects of renal Ca reabsorption on measured δ^44/42^Ca_urine_. In order to assess whether such a common BDC baseline approach actually works, many more data will be needed. Furthermore, the influence of dietary Ca isotopic composition on δ^44/42^Ca_urine_ and other potential fractionation mechanisms in the body have to be better understood, and represent significant gaps in our present knowledge that must be addressed in future studies.

## Summary and Conclusion

We present time series of Ca isotopic compositions in urine from seven individuals participating in a 21-day bed rest study. Our results document systematic temporal variations in the isotopic composition of excreted calcium. As bed rest progresses, the Ca isotopic composition of urine is shifted toward “lighter” values compared to pre-bed rest compositions. This can be explained by an increased flux of isotopically light Ca entering the blood from bone dissolution, and thus these shifts may be a useful indicator of bone loss. Statistical analyses of the data show a time delay of 14 to 21 days before the shifts in urine Ca isotopic composition become apparent. This finding is consistent with time delays observed in biochemical markers of bone’s organic matrix, as well as in bone densitometric measurements.

The data show huge differences in δ^44/42^Ca_urine_ between individuals of up to 0.8‰. These differences are most likely caused by each subject’s unique Ca metabolism, and especially the percentage of Ca being reabsorbed in the kidneys. Small differences in the renal Ca reabsorption rate can easily explain the magnitude of the observed inter-individual δ^44/42^Ca_urine_ shifts. The fact that the amounts of Ca excreted daily are well correlated with δ^44/42^Ca_urine_ implicates renal Ca reabsorption rate as the cause of these differences.

The individuality in Ca metabolism hampers the use of δ^44/42^Ca_urine_ in a straightforward manner for determining net bone mineral balance or detecting disorders in Ca metabolism. This problem can be overcome if a δ^44/42^Ca_urine_ baseline can be determined for the individual prior to bone loss. Then, changes in δ^44/42^Ca_urine_ from the baseline can be directly related to the net bone mineral balance and/or Ca metabolism.

An extremely important finding is that the data on all seven subjects, taken together, show a strong correlation between the total amount of excreted Ca and Ca isotopic composition of urine. We observe that δ^44/42^Ca_urine_ is lower during the bed rest phase compared to pre-bed rest phase δ^44/42^Ca_urine_ for an identical amount of excreted Ca. This suggests that it may be possible to use data on δ^44/42^Ca_urine_ and amounts of urinary Ca excreted from healthy persons to construct a normal bone balance baseline for the general population. In this case, offsets from the baseline would be diagnostic of disorders in Ca metabolism; in particular, individuals with net bone loss would be expected to exhibit lower δ^44/42^Ca_urine_ for a given amount of excreted Ca relative to the baseline.

## Data Availability

The datasets generated for this study are available on request to the corresponding author.

## Author Contributions

AH conducted the chemical preparation of the samples, did the mass spectrometry work, and wrote the first version of the manuscript. PF-M did the sample collection and contributed to the methods section of the paper. JR contributed to the discussions section and did the statistical analysis of the time delays. SG conceived the mass spectrometry, calibrated the double spike, performed the Ca isotopes data reduction, contributed to the discussion section, and did linguistic revisions of the manuscript.

## Conflict of Interest Statement

The authors declare that the research was conducted in the absence of any commercial or financial relationships that could be construed as a potential conflict of interest.

## Footnotes

1 http://www.r-project.org
